# Microhardness and Microstructure Analysis of the LPBF Additively Manufactured 18Ni300

**DOI:** 10.3390/ma17030661

**Published:** 2024-01-29

**Authors:** Pablo M. Cerezo, Jose A. Aguilera, Antonio Garcia-Gonzalez, Pablo Lopez-Crespo

**Affiliations:** Department of Civil and Materials Engineering, University of Malaga, C/Dr Ortiz Ramos, s/n, 29071 Malaga, Spain; pm@uma.es (P.M.C.); j.a.aguilera@uma.es (J.A.A.); tolin@uma.es (A.G.-G.)

**Keywords:** 18Ni300, additive manufacturing (AM), laser powder bed fusion (LPBF), microhardness, micro-indentation, microstructure

## Abstract

This research focuses on analysing the 18Ni300 maraging steel produced through laser powder bed fusion. Specifically, it aims to examine the phase components using X-ray diffraction, the microstructure through scanning electron microscopy, and the hardness of the different structures present in the manufactured material. The primary goal is to meticulously analyse the material and its microstructures. By doing so, a correlation between the hardness and each structure type, be it cellular or columnar, can be established. This will allow us to pinpoint any defects in the material before any surface chemical treatment is carried out and facilitate a thorough examination of its microstructure. A consistent pattern emerges across the samples through systematic measurement of microhardness distribution in various locations and detailed examination of the structure. The findings of the study reveal that the hardness of cellular and columnar structures exhibits a significant variation based on the location of the measurement about cell boundaries. The hardness value is notably higher in the combination of cellular and multiple layers, as the data indicate.

## 1. Introduction

Laser Powder Bed Fusion (LPBF) is a widely used powder bed-based additive manufacturing (AM) technique due to its advantages over conventional manufacturing methods, such as machining and forging. These include the ability to create complex shapes used extensively for weight-saving topology optimisation [1,2] that has so far been constrained by the limitations of tool accessibility [3,4], as well as meet the demand for intricate and complex moulds and dies which have grown with the advancements in manufacturing technology. A new range of shapes can be manufactured in short periods with no additional cost or energy compared to traditional forms. Additional benefits include the reduction of manufacturing steps and low material wastage [3,4,5,6].

AM has become a popular choice for metal parts manufacturing with the ability to create parts with near-net shape quality and intricate geometries with sizes as small as 100 μm [7]. Processes such as LPBF, electron beam melting, and binder jetting are utilised for AM [8,9].

The characteristics of pieces produced by LPBF exhibit noticeable distinctions from their counterparts made via conventional manufacturing methods. The most important difference when using LPBF is the presence of porosity that can be significant when building parameters are not correctly optimised. The microstructure presented in this method is quite complex as it does not follow the conventional solidification process, where only one solidification process occurs. Due to the melting of the material after the deposition with the next layer, the microstructure presents a finer microstructure in several metals [5,10,11,12,13,14,15], including maraging steels [16] and grains. LPBF is also known as Selective Laser Melting (SLM), a powder bed fusion process type, ISO/ASTM 52900:2023 standard [17].

The presence of pores within a material significantly reduces its mechanical properties, including strength and fatigue performance [18]. This effect arises from the tendency for pores to facilitate crack propagation, particularly in cases where the pores are irregular or clustered, leading to high-stress concentrations. It is well established that the incorporation of pores within the structure of a material has the potential to reduce its effective load-bearing cross-sectional area significantly [19]. Conversely, the LPBF material’s microstructure is inherently fine, and it has been demonstrated that such a property confers elevated levels of hardness and strength without the need for post-manufacture heat treatments [10], depending on the specific alloy. A common drawback is its relatively low ductility [5].

Maraging steels are highly valued for their outstanding mechanical properties, which include excellent ductility, high yield strength, remarkable mechanical resistance, and super weldability [20,21,22,23]. Unlike high-carbon Ni-based alloys, Fe-based alloys, and Al-based alloys, maraging steels are low-carbon martensitic non-stainless steels less prone to cracking during rapid cooling and solidification [24,25,26,27,28]. These exceptional characteristics make maraging steels ideal for tooling materials utilised in extreme conditions like injection moulding, die casting, hot-pressing, and severe plastic deformation processing. Furthermore, their ability to resist thermal cracking during rapid cooling makes them a preferred material for AM, where high dimensional stability is of utmost importance [28,29,30].

The LPBF process produces an ultrafine cellular solidification structure with high dislocation density due to the rapid solidification process 18Ni300, a martensitic matrix that characterises the microstructure. Intercellular microsegregation typically leads to the chemical stabilisation of retained austenite (RA) at the boundaries of cells, with a potential volume fraction of up to 11% [19]. The microstructure that has been produced exhibits an entirely different composition from that of the conventionally processed 18Ni300 counterpart. Precisely, it displays a fully martensitic microstructure in the solution-annealed state. If 18Ni300 is directly aged after LPBF, it contains more austenite than its solution-annealed and aged counterpart (RA+ reverted austenite during ageing) [31].

The strength of age-hardened LPBF 18Ni300 is comparable to conventionally processed parts, indicating that higher vol% of softer austenite does not significantly affect tensile strength [19]. However, mechanical properties such as ductility and tensile strength can be reduced compared to forged “solution-annealed and aged” counterparts by the presence of porosity through different building orientations [32,33].

The rapid heating and cooling rates involved in laser processing often result in high residual stresses in LPBF-produced components. These residual stresses, in turn, can lead to stress cracking and interlayer debonding. Residual stresses in LPBF-processed samples have high tensile stress near the boundaries and intermediate compression stress in the centre zones [15]. The control of stress levels in laser processing relies on carefully managing processing parameters and substrate preheating to reduce temperature gradients [34]. After manufacturing, materials may contain residual stress levels that can be eliminated or reduced by post-fabrication heat treatments [35].

Despite the high density achievable through LPBF technology, which can reach rates of approximately 99%, residual porosity in LPBF-fabricated parts poses a significant challenge for their practical utilisation in high-strength and fatigue-resistant applications [36]. Components manufactured using LPBF have mechanical properties influenced by the resulting microstructure, similar to conventionally manufactured parts [37,38]. Understanding the microstructure and microhardness of each of the structures in the material has been a critical aspect in establishing the mechanical properties of the final parts.

Numerous studies [16,39,40] have measured the hardness of samples using a large load, which results in a significant footprint that considers various structures and grains to obtain an average hardness of the sample. However, this research is based on the use of micro-indentation to study the microstructures individually, cellular and dendritic, and the influence of welding path on the microstructure hardness.

To observe the microstructure of a material, it is necessary to chemically attack it, so pores and other imperfections are also modified. Because of this, it is not possible to measure dimensions after the attack, but it is possible to know the position. Hence, this study aims to make a correlation between position and microstructure. Understanding the differences in the hardness of the microstructure, it is possible to know in which situation these imperfections would be found before attacking the surface and seeing the grains or the existing structures.

On the other hand, if the surface does not undergo any chemical attack, it may be challenging to identify the location of the pores in the microstructure. Still, the dimensions of these pores remain unaltered. This study aims to understand the hardness in each area, establishing a new relation to predict the microstructure before a chemical attack.

## 2. Material and Methods

The LPBF process uses as a base material 18Ni300 powder particles with a diameter of 40 µm. It has a well-defined chemical composition, as presented in Table 1, provided by the material supplier.

**Figure 1:**
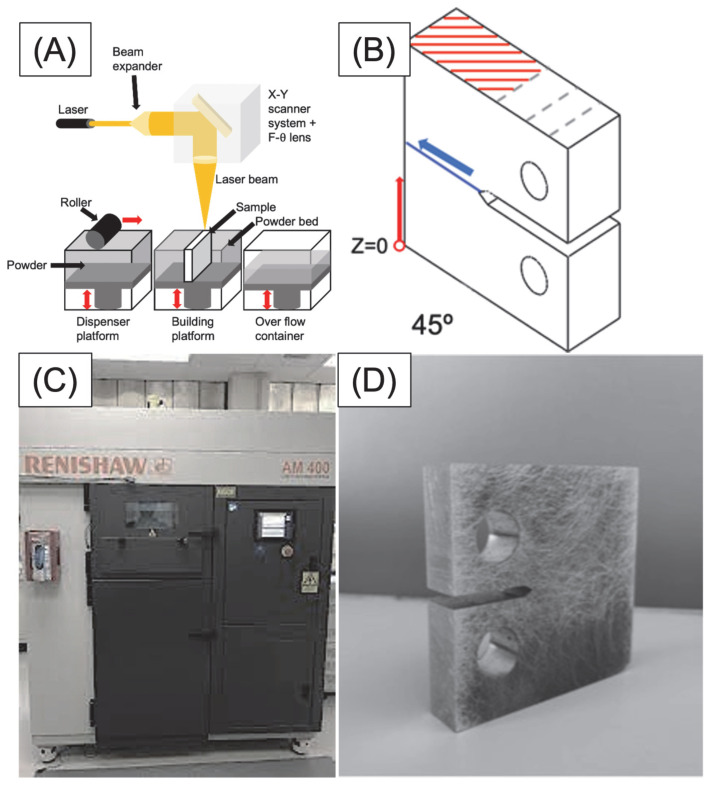
(**A**) Schema of LPBF process and (**B**) LPBF layer orientation pattern (red lines are weld orientation, the blue line is the crack plane, and the blue arrow is the direction of the crack). (**C**) Renishaw AM 400 LPBF equipment used [41] and (**D**) 45° 18Ni300 sample manufactured.

The current study employed the LPBF process to produce compact tension (CT) specimens by melting steel powder particles layer by layer using a high-power laser via Lasercusing^®^ (Figure 1A). The Renishaw (Wotton-under-Edge, UK) equipment model “AM 400” was utilised for the LPBF processes (Figure 1C). The process parameters were optimised to achieve the desired results. A laser power of 400 W was employed with a scan speed of 0.85 m/s and a laser diameter of 0.04 mm. The deposited layer had a thickness of 30 µm, while the hatch spacing was 100 μm with 25% overlapping.

The CT specimens were manufactured with an orientation of 45° with respect to the notch where the crack starts in a uniaxial fatigue test. The impression was produced layer by layer, coinciding the layers with the crack propagation plane and their parallels, as shown in Figure 1B. The building direction in the LPBF, the pattern illustrated in Figure 1B, was followed from z = 0 to z = 60 mm, and the notch and the holes were machined. This particular angle was selected based on research findings that indicate lower fatigue resistance at 45° angles compared to 0° or 90° printing orientation. After fabrication, any post-treatment was applied.

Before performing any examination, the specimens were sectioned in a parallel plane to the surface of the specimen. The samples were meticulously prepared in adherence to the well-established metallographic practice specified in ASTM E407-99 [42]. Then, the material was separated into two batches. Both of them underwent a chemical attack for 70 s using Nital, a solution of nitric acid with a concentration of 4%, and ethyl alcohol. This procedure aimed to ascertain the microstructure and morphology of the grains using an optical microscope (OM), the Leica DM 6 M OM (Wetzlar, Germany). Density measurements were performed by taking an image of the whole surface through the OM in bright field and using ImageJ v1.53t to calculate the percentage of surface area excluding gas pores and lack of fusion, obtaining a porosity of 0.187% of the total area [43].

The same sample microstructure was analysed through scanning electron microscopy (SEM) CLARA-TESCAN (Brno, Czech Republic), using energy applied 20 keV, an intensity of 300 pA and a magnification between 3.5 kx and 43.5 kx. The composition of the phases was analysed using X-ray diffraction (XRD) with CuKα1 radiation on a Bruker D8 DISCOVER diffractometer (Madrid, Spain). The measurements ranged from 10° to 90° (2θ) for 120 min with a step size of 0.0167. The sample was not spun to prevent any precision effects, and the tube operated at 40 kV and 40 mA. A Scintillation detector collected the diffracted beam.

**Figure 2:**
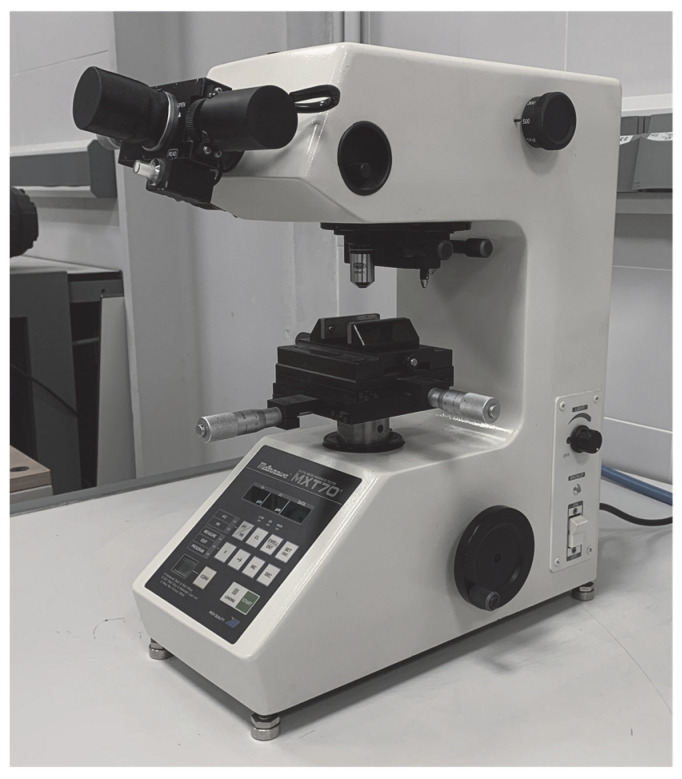
Micro-indentation tester Matsuzawa MXT70 used for Vickers microhardness.

The same cross-sections studied in the SEM and EDX were analysed with Vickers microhardness (HV) using the MXT70 Tester (Matsuzawa, Nagano, Japan) (Figure 2). The indentations were spaced 200 µm apart, and a load of 200 gf was applied with a 10 s dwell time, according to ASTM E384 [44]. The measurements were conducted in an 8 × 8 grid template. After the test, the samples were examined with the OM used before to measure the size of the mark. The data analysis was performed using MATLAB R2022b.

## 3. Results and Discussion

### 3.1. Microstructure

**Figure 3:**
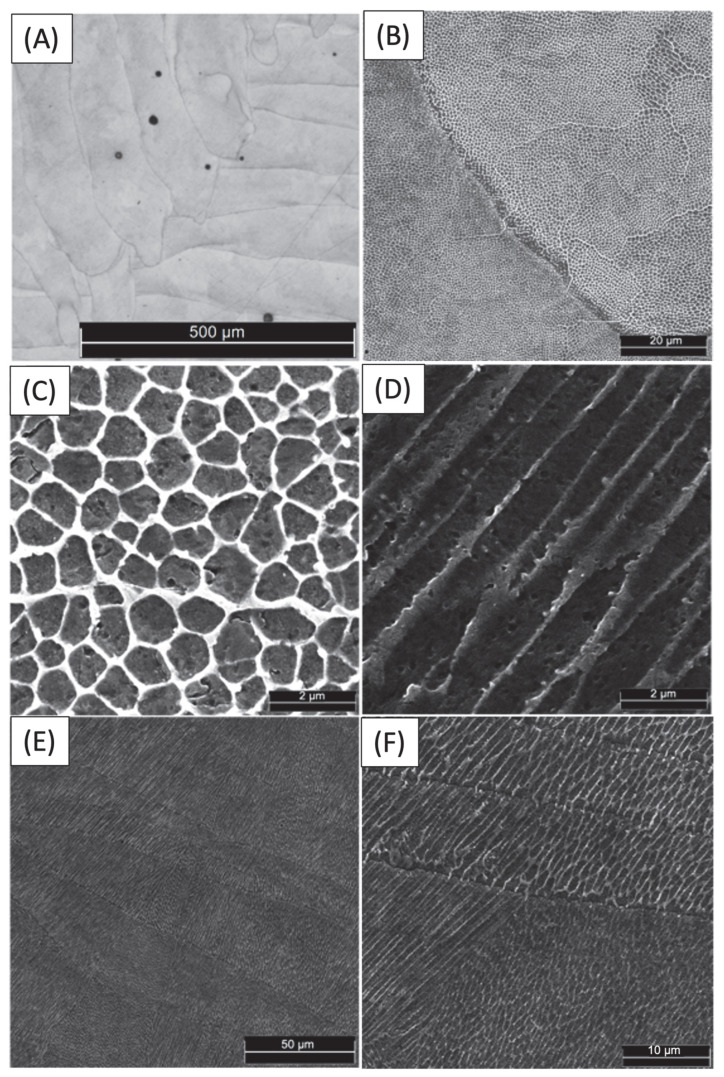
Microstructure of 18Ni300 maraging steel after etched with Nital for 1 min. (**A**) Optical micrograph, (**B**) SEM micrographs showing the grain boundaries and the welding paths, (**C**,**D**) high-magnification SEM micrographs showing the cellular dendrites of LPBF-processed parts, and (**E**,**F**) high-magnification SEM micrographs showing grains across different welding paths.

The OM and SEM micrographs of a section of the LPBF 18Ni300 sample, after being etched with 4% Nital for 70 s, are shown in Figure 3. This sample section is the same as the one where the micro-indentation is performed. In the LPBF process, the cooling rate is inconsistent throughout the melting pool, so the perimeter of the melting pool undergoes the highest cooling rate, resulting in quicker solidification compared to the inner regions. Consequently, the cooling rate leads to segregation or a change in the dendritic or cellular substructure, resulting in the visibility of the melting pool boundaries post-etching [45].

Figure 3A shows the melting pool boundaries and boundaries of the welding path in a layer with distinct colour differences. The effect is more pronounced in Figure 3B. The welding paths exhibit a discernible degree of elongation and are characterised by an approximate width of 180 µm. The grain structure is depicted in Figure 3B,E,F. It can be observed that the exact grain passes through multiple weld paths instead of remaining within them. The observed phenomenon may be attributed to the intricacies of the manufacturing process, during which the base layer undergoes a reheating process upon the application of the top layer. This results in the continued growth of the grain along the same orientation as the previous layer, thereby yielding a novel material with comparable properties [46]. The results of the density measurement indicate that the relative density of the LPBF maraging steel was determined to be 99.85%. This value is near the fully dense bulk maraging steel, suggesting that the LPBF process produced a material with a high level of density.

Figure 3E,F shows that grain growth in the newly provided material was consistent despite varying welding paths. However, there are slight differences in the network parameters of the dendritic cell structure on some occasions, as can be observed in Figure 3F.

In theory, the expected growth of cellular dendrites is perpendicular to the melt pool boundary, as heat flux typically runs along the fusion line’s normal. However, in practice, the growth orientation of dendrites and the tangential direction of the melt pool boundary often deviate from a 90-degree angle due to the influence of the preferred crystal structure growth orientation, in addition to the heat flux direction [47].

Micrographs in Figure 3C,D show cellular and dendritic solidification morphology and epitaxial grain growth on a section perpendicular to the layer build sequence. Upon examination, it is evident that the as-fabricated LPBF maraging steel exhibits a very fine cellular structure with an intercellular spacing of 0.93 µm. The high cooling rate of the melting pool prevents the formation of secondary dendrite arms, resulting in a fine microstructure [47]. It is noteworthy that the cellular structure, being three-dimensional, can exhibit both equiaxed and columnar morphologies when viewed in a two-dimensional plane, depending on the observer’s angle of view. LPBF steels typically feature fine cellular structures, leading to superior hardness and strength compared to conventionally manufactured steels [48]. Other researchers have validated these statements in direct metal laser sintering of maraging steel 300, processed on an EOS machine [28,49]. The intercellular spacing can change between welding paths due to varying heat flux direction and intensity caused by differences in grain surroundings.

**Figure 4:**
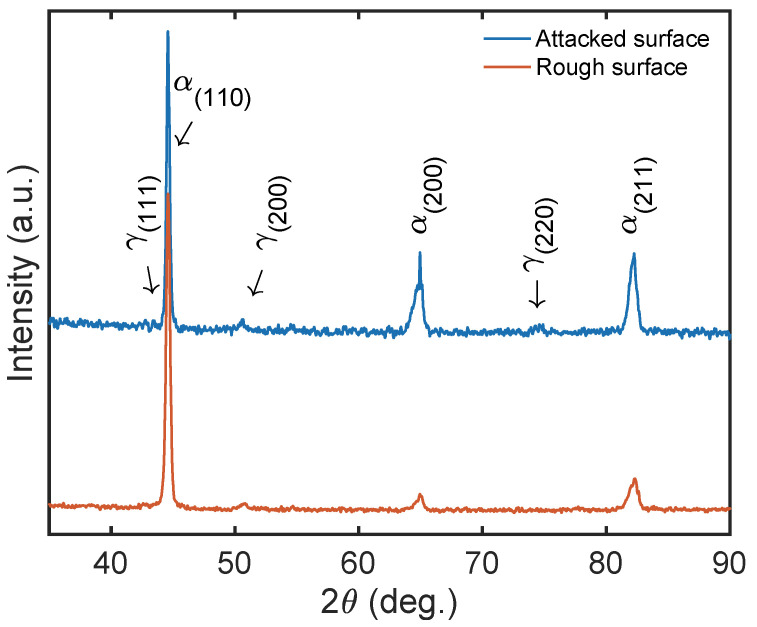
LPBF processed the XRD pattern of the 18Ni300 parts before and after the attack with 4% Nital for 1 min.

Based on the observations in Figure 3, it seems that the colour of the boundaries separating cellular and dendritic structures may not match the structures themselves. This inconsistency may be attributed to the potential presence of RA, which has been observed in previous studies [19]. To validate the presence of RA within the system, an XRD phase analysis was conducted (Figure 4).

### 3.2. Phase Analysis

XRD results for 18Ni300, including rough and attacked surfaces, were analysed in Figure 4. The LPBF-manufactured components’ pattern was observed across a broad range of 35°–90° with varying ω, displaying robust diffraction peaks that corresponded to the crystal planes (110), (200), and (211) of martensite (α-Fe). The fabricated component was predominantly comprised of a martensitic phase, with a minor proportion of austenite phase (γ-Fe), with slight contribution in the crystal planes (111), (200), and (220) [50].

The topographical disparity between the rough and attacked surface is exhibited through the peaks corresponding to austenite’s presence. Notably, the austenite at the edges of the welding paths is concealed until the surface is subjected to chemical treatment, thereby revealing the edges between the beads, and then presenting a no-null intensity when an XRD test is performed on the attacked surface. The formation of RA is caused by the segregation of solute elements, primarily nickel, at cellular boundaries during solidification. The inclusion of nickel in the alloy composition serves to stabilise the RA, thereby promoting the facile detection of the austenite phase [50,51], as seen in (111), (200), and (220).

### 3.3. Microhardness

Compared to conventional hardness tests, the results of a footprint test are significantly more precise. They can accurately detect various phases and structures in the material as it can be done in only one structure. The smaller footprint size obtained from this test enables a superior level of precision compared to the average measurements obtained from a high-load hardness test.

The presence of different structures (cellular and columnar) in the building layers can result in a variation of the hardness. The discrepancy in each measurement indicates the anisotropy inherent in LPBF, a well-known attribute of AM techniques utilised to produce metal components [52,53,54,55]. The phenomenon can be attributed to the layer-wise build approach and the utilisation of the “island” scan strategy in AM processes [56]. This methodology leads to localised melting of powder particles, resulting in the development of non-homogeneous morphologies and anisotropic grain structures, which have been well-documented in the academic literature [57,58].

**Figure 5:**
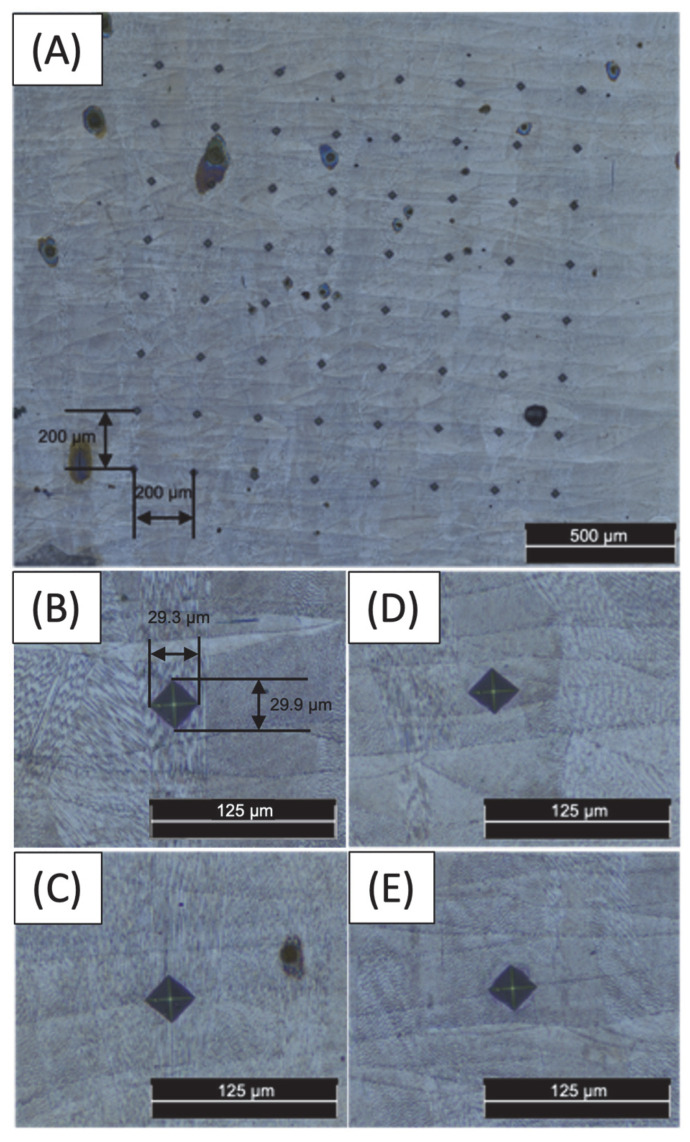
Images of 8 × 8 pattern micro-indentations on an etched surface. (**A**) General view, (**B**) on a single layer with a columnar structure, (**C**) on several layers with a columnar structure, (**D**) on a single layer with a cellular structure and (**E**) on several layers with a cellular structure.

The present study involved obtaining measurements in an 8 × 8 pattern of an etched surface. This was performed to determine the specific structure under investigation, as depicted in Figure 5A. A closer view of the micro-indentation in columnar structures is shown in Figure 5B,C, and according to the results in Figure 5B, a single columnar structure exhibited a hardness of 314 HV. Interestingly, when the hardness in the joints between multiple columnar structures was measured, a higher value of 323 HV appeared. This observation can be attributed to the presence of RA in the structure boundaries, the result of the segregation of solute elements, mainly nickel, at the boundaries of cells during the process of solidification, which had higher values than the other areas [50].

The micro-indentations of cellular structures are illustrated in Figure 5D,E. In Figure 5D, a unique cellular structure was evaluated, yielding a hardness value of 317 HV. However, when assessing the hardness of numerous cellular structure joints, the value rose to 333 HV. This augmentation is akin to that witnessed in the columnar structure and is attributed to the same factor.

Comparing the cellular and the columnar structures, it was observed that cellular structures show a higher microhardness attributed to the fine-grain microstructure, resulting in a higher dislocation density of austenite cells, shown in AM 316L SS samples [59]. The prevention of slip motion along the grain boundaries increases strength and resistance to deformation.

**Figure 6:**
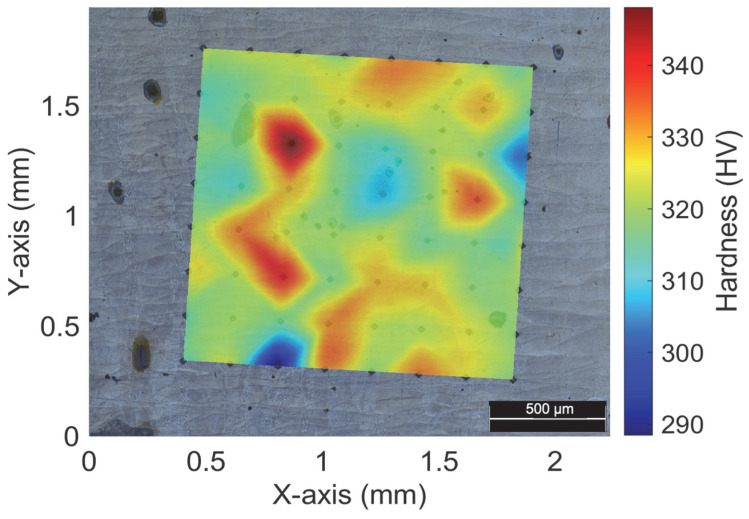
Etched surface image with overlaid hardness map and measured values.

**Figure 7:**
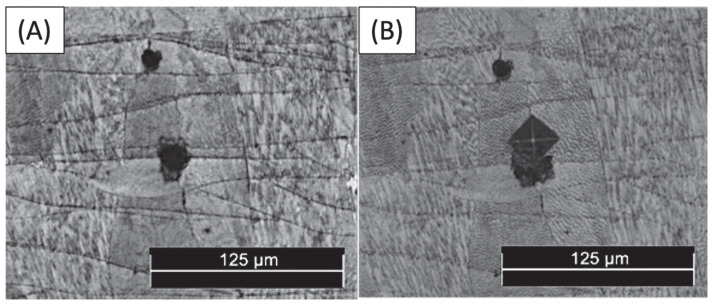
(**A**) Lack of fusion previous to the microhardness test, and (**B**) micro-indentation on a lack of fusion.

Figure 6 showcases a map that exhibits the microhardness readings acquired from every point on the grid. The values are mostly uniform, with the existing structures, cellular and columnar. The micro-indentations depicted in x = 0.8 mm and y = 0.35 mm show a minimum of 288 HV that could be considered an outlier that distorts the rest of the measurements. In Figure 7A,B, the reason for this value is highlighted.

Wrought maraging steel tends to exhibit a hardness level ranging from 285 to 351 HV [60,61], even without undergoing ageing heat treatment—a finding that aligns with the measurements illustrated in Figure 6. The highest level of hardness is achieved when the material features a cellular structure, whereas the lowest level of hardness is observed when there are measurement defects present. By implementing ageing heat treatment, such defects can be minimised, resulting in an enhancement of the material’s mechanical behaviour—including an increase in its hardness level, among other benefits [60,62].

The impact of porosity or lack of fusion on the micro-indentations can be observed in certain areas due to the proximity of these defects. As illustrated in Figure 7B, when a micro-indentation was performed on a lack of fusion and its surrounding region (Figure 7A), a significant reduction in microhardness (down to 288 HV) was observed, in what should have been an increase in hardness due to the existence of numerous cellular structure joints. The absence of martensitic phases is the root cause of this phenomenon, as it results in a dearth of structural links. Other variations have not been detected, which is akin to the outcomes of [56]. In that particular research, there was a decline in dislocation levels at the subgrain boundaries and inclusions, which corresponded with the microhardness. According to the findings illustrated in Figure 6, it is evident that the variability of the measurements was significantly reduced. This is supported by the observation that the maximum and minimum values of the measurements are proximate, except for the 288 HV value.

## 4. Conclusions

In conclusion, the current study has provided valuable insights into the microstructure and microhardness of 18Ni300 fabricated via LPBF. By examining the relationship between the structures, their composition, and their effect on hardness, we were able to identify a direct influence of the cellular and columnar structure on the hardness values obtained. The findings of this study have significant implications for future research in this area and are expected to contribute to the development of more effective and efficient manufacturing processes for 18Ni300 and other similar materials. After performing a comprehensive investigation, the research findings led to the following conclusions:

(1) Cellular structures present a higher hardness than columnar structures due to the higher presence of dislocation of austenite cells attributed to the fine-grain microstructures.
(2) The enhanced strength of joined structures compared to that of a single structure was conclusively attributed to nickel segregations at the edges of the structures. This finding is based on the existence of RA in the structure boundaries, which can be attributed to the segregation of solute elements, mainly nickel, at the boundaries of cells during solidification. The hardness between joined structures and single structures increases by 3–5%.
(3) The absence of fusion causes a reduction in the hardness of the material in that region owing to the absence of martensitic phases and structural connections. This phenomenon is attributed to the inability of the material to achieve its full strength potential due to the compromised microstructure in the affected area.

Further research is required to investigate and comprehend the dislocation concentrations around imperfections, including the lack of fusion and the gas porosity. Forthcoming work will concentrate on precipitation behaviour and reverse austenite nucleation utilising transmission electron microscopy (TEM) and electron backscattered diffraction (EBSD).

## Figures and Tables

**Table 1:** Chemical composition of 18Ni300 maraging steel.

wt/%	Fe	Ni	Co	Mo	Ti	Cr	Si	Al	Mn	C	P
18Ni300	Balance	18.2	9.0	5.0	0.6	0.3	0.1	0.05	0.04	0.01	0.01

## Data Availability

Data are contained within the article.
